# INVESTIGATING MEDIATORS OF SYSTEMIC MITOCHONDRIAL BIOENERGETICS IN COGNITIVE IMPAIRMENT AND ALZHEIMER’S DISEASE

**DOI:** 10.1093/geroni/igae098.2982

**Published:** 2024-12-31

**Authors:** Gargi Mahapatra, Jaclyn Bergstrom, Antonio Michel Pinto, Jolene Diedrich, Suzanne Craft, Anthony Molina

**Affiliations:** University of California San Diego, La Jolla, California, United States; UC San Diego, La Jolla, California, United States; Salk Institute for Biological Studies, San Diego, California, United States; Salk Institute for Biological Studies, San Diego, California, United States; Wake Forest University School of Medicine, Winston-Salem, North Carolina, United States; University of California San Diego, La Jolla, California, United States

## Abstract

We previously showed peripheral blood mononuclear cell (PBMC) and platelet mitochondrial bioenergetic capacities are lower in older adults with Alzheimer’s disease (AD), with notable changes in fatty acid oxidation (FAO)-mediated respiration. The metabolic pathways underlying mitochondrial functional differences remain un-identified. This study investigated lipid metabolites that are differently expressed across different stages of cognitive impairment (CI). Targeted lipidomics analyzed 51 fatty acids (FAs) in PBMCs and platelets of older adults with normal cognition (NC), mild cognitive impairment (MCI), and AD dementia (DEM). 10 FAs were selected in each cell type based on significant concentration differences between different cognitive groups. In PBMCs, 9 FAs exhibited higher concentrations in MCIs than NC and DEM, with significantly lower concentration in DEM than MCI, while 1 FA exhibited progressively declining concentrations from NC to MCI to DEM. Significant positive correlations were observed between FA concentrations and FAO and maximal mitochondrial respiration (Max). In platelets, 7 FAs showed progressively declining concentrations from NC to MCI to DEM, and significant positive correlations with FAO and Max, while 3 FAs showed progressively increasing concentrations with cognitive impairment, and significant negative correlations with FAO and Max. Significant negative correlations were also observed with mPACC5 cognitive assessment scores and hippocampal volumes. Pathway analyses identified FA biosynthesis, alpha-linolenic acid, and alpha-linoleic acid metabolism as involved pathways differently impacted across cognitive groups. These findings emphasize the importance of FAs and related metabolic pathways in systemic bioenergetic decline in CI, and may facilitate development of preventative, diagnostic, and therapeutic approaches in AD.

